# MiR-18a and miR-17 are positively correlated with circulating PD-1^+^ICOS^+^ follicular helper T cells after hepatitis B vaccination in a chinese population

**DOI:** 10.1186/s12865-018-0263-y

**Published:** 2018-07-28

**Authors:** Xiaojia Xu, Yulian Li, Yaping Liang, Mingjuan Yin, Zuwei Yu, Yan Zhang, Lingfeng Huang, Jindong Ni

**Affiliations:** 1 Department of Environmental and Occupational Health, Dongguan Key Laboratory of Environmental Medicine, School of Public Health, Guangdong Medical University, Dongguan, 523808 China; 2 Dalang Community Health Service Centers, Dongguan, 523770 China

**Keywords:** Follicular helper T cells, MiR-17–92, Hepatitis B vaccine

## Abstract

**Background:**

While vaccination remains the most effective method to control hepatitis B virus (HBV) infection, 5–10% of recipients exhibit non-responsiveness to the HB vaccine. Immunological analysis of strong, weak or absent protective antibody responses to the HB vaccine should provide insights into the mechanisms that contribute to non-responsiveness.

**Results:**

We investigated the potential involvement of follicular helper T (Tfh) cells in the immune response to HB vaccine, and associations between the miR-17–92 cluster and Tfh cells. We recruited 12 adults who had completed the HB vaccination course during childhood. Following a booster dose of HB vaccine, hepatitis B surface antibody (HBsAb) titers, percentage of PD-1+ICOS+ circulating Tfh (cTfh) and plasma cells, and expression of miR-17–92 were assessed at baseline (before immunization) and after vaccination on days 7 and 14. Notably, the HBsAb level gradually increased after HB vaccination while the proportion of PD-1+ICOS+ cTfh cells was significantly increased on day 7 relative to baseline, so as plasma cells. Expression of miR-18a and miR-17 within the miR-17–92 cluster and HBsAb titers in CD4+ T cells were positively correlated with the PD-1+ICOS+ cTfh cells proportions after HB vaccination.

**Conclusions:**

The increase in HBsAb titers was positively associated with expression of all the components of the miR-17–92 cluster except miR-19a. Our findings indicate that the miR-17–92 cluster contributes to antibody production, and miR-18a and miR-17 are involved in Tfh cells differentiation after HB vaccination.

## Background

Hepatitis B is a major public health priority worldwide with more than 350 million chronic hepatitis B virus (HBV) carriers, accounting for approximately 686,000 deaths per year [1]. The hepatitis B (HB) vaccination is currently the most effective and economical measure to prevent HBV infection. However, 5–10% patients exhibit non-responsiveness to the vaccine and remain susceptible to infection. Immunological assessment of strong, weak or absent protective antibody responses to the HB vaccine should provide further insights into the precise mechanisms underlying non-responsiveness to hepatitis B vaccine.

CD4^+^ T cells play a crucial role in assisting B cells with antibody production. T helper (Th) cells, mainly Th1 and Th2, are widely recognized as key cells that regulate the immune response to HB vaccine whereas Th cells play a regulatory role only in B cell activation [2, 3]. Recent studies have demonstrated that the follicular helper T (Tfh) subset of CD4^+^ T cells plays a major role in B cell-mediated production of high-affinity, class-switched antibodies, and generation of high-affinity memory B cells through germinal center (GC) reaction during infection and vaccination [4, 5]. The transcriptional repressor, Bcl-6, is essential for Tfh cell differentiation and IL-21 induces B cell proliferation and differentiation [6]. Tfh cells are characterized by expression of chemokine (C-X-C) receptor 5 (CXCR5), co-stimulatory molecules (ICOS) and programmed death 1 (PD-1), which are critical for their function [7]. CXCR5^+^CD4^+^ T cells in peripheral blood are known as circulating Tfh (cTfh) cells. In healthy adults subjects cTfh cells resemble Tfh cells in their capacity to produce IL-21 and induce B cell differentiation [8–10]. Researchers generally utilize cTfh for study instead of Tfh due to the difficulty in obtaining lymphoid T cells in humans. Accumulating studies suggest that the antibody response to vaccination in humans is correlated with cTfh cells [11, 12]. However, the issue of whether Tfh cells are involved in immune response to the HB vaccine is unknown at present.

MiRNA-mediated post-transcriptional regulation plays a key role in the differentiation and function of Tfh cells [13]. Tfh cells display a characteristic miRNA expression profile, compared to other effector Th cells [6, 14] and differentiation fails in the absence of miRNA [15]. Several miRNAs and miRNA clusters reported to be involved in the regulation of Tfh cells [16]. Recent progress in clarifying the mechanisms by which Tfh cells are affected has revealed important roles of posttranscriptional control of gene expression mediated by miRNAs, including miR-17-92, miR-155 and miR-146a [17, 18]. The miRNA cluster, miR-17–92, is encoded by a polycistronic miRNA that generates a single precursor transcript for six distinct mature miRNAs: miR-17, miR-18a, miR-19a, miR-19b-1, miR-20a and miR-92a-1 [19]. Expression of miR-17–92 is induced early during T cell activation and suppressed after completion of Tfh cell differentiation [20], suggesting a role in the immune response to HB vaccine.

To explore the pathways underlying the immune response to HB vaccine, we investigated the involvement of cTfh cells. Furthermore, we identified the relationship between miR-17–92 and Tfh cells after HB vaccination. Data from our study provide novel insights into the roles of miR-17–92 and cTfh cells in the immune response to the HB vaccine.

## Results

### Dynamic changes in HBsAb titers, PD-1^+^ICOS^+^ cTfh cells, plasma cells and miR-17–92 after HB vaccination

**Fig. 1:**
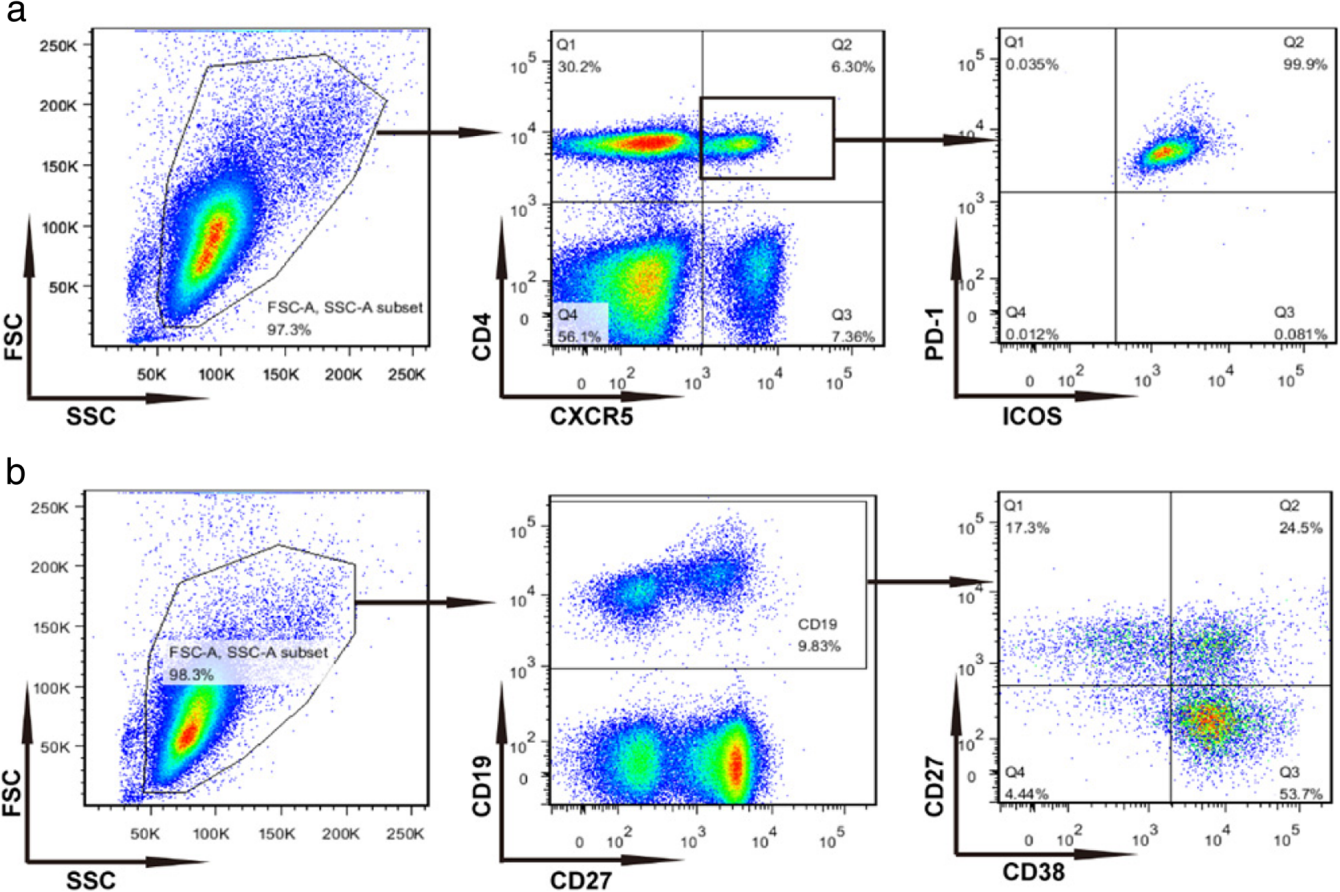
Gating strategy for the analysis of cTfh cells and plasma cells. **a** Representative flow cytometry plots from PBMC showing the gating scheme for isolating T cell. **b** Representative flow cytometry plots from PBMC showing the gating scheme for isolating B cell

**Fig. 2:**
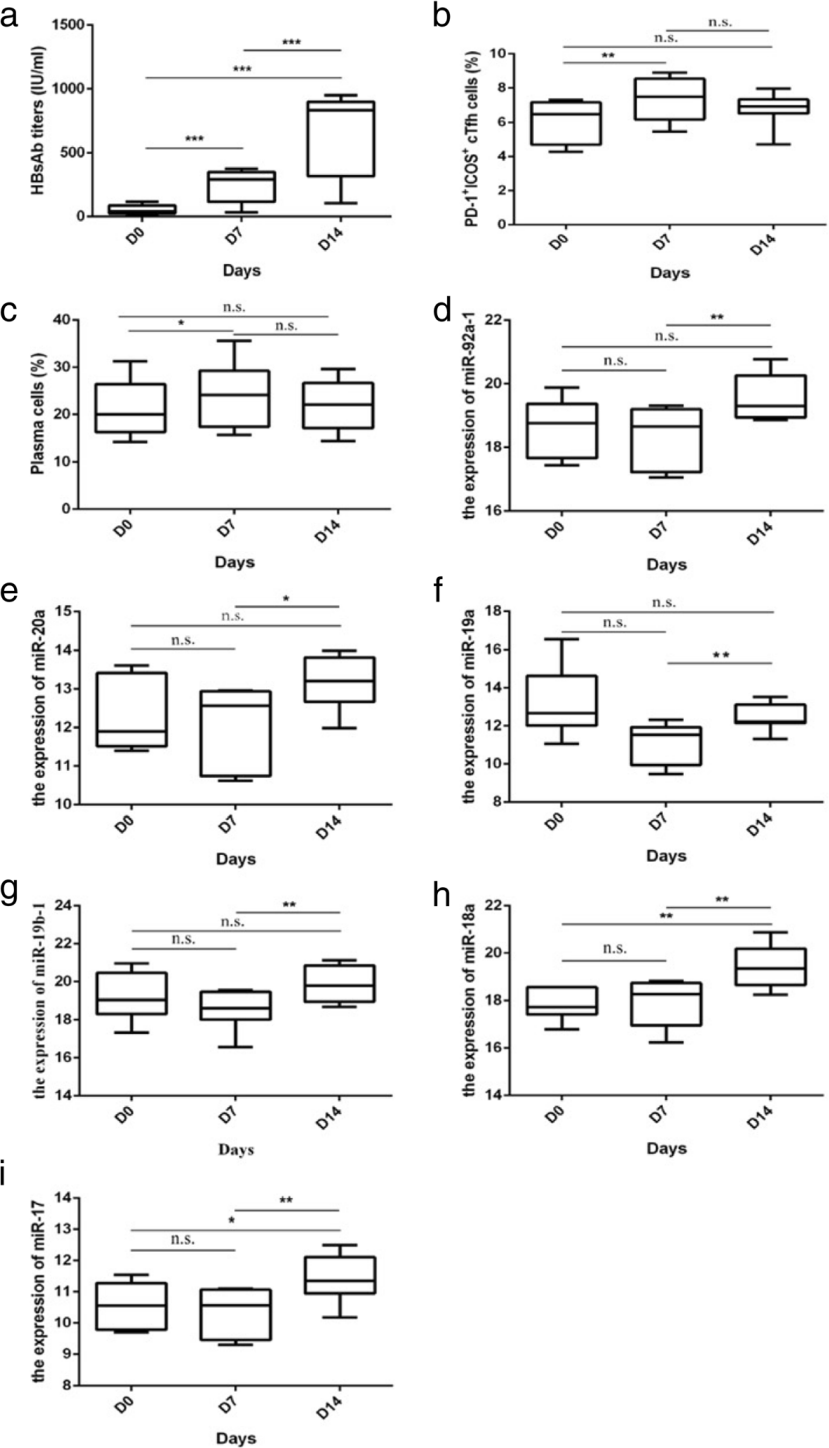
Dynamic changes of HBsAb titers, PD-1^+^ICOS^+^ cTfh cells, plasma cells and miR-17~ 92 after HBV vaccination. **a** The level of HBsAb titers in different points (day 0, 7, 14). **b** The percentage of PD-1^+^ICOS^+^ cTfh cells. **c** The percentage of plasma cells. **d** The level of miR-92a-1 in CD4^+^ T cells. **e** The level of miR-20a in CD4^+^ T cells. **f** The level of miR-19a in CD4^+^ T cells. **g** The level of miR-19b-1 in CD4^+^ T cells. **h** The level of miR-18 in CD4^+^ T cells. **i** The level of miR-17 in CD4^+^ T cells.*, *P* < 0.05; **, *P* < 0.01; ***, *P* < 0.001; n.s., no significant differences

Serum samples were obtained from 12 adult subjects at baseline, 7 and 14 days after HB vaccination. The gating strategy for analysis of cTfh and plasma cells is shown in Fig. 1. We observed alterations in the level of HBsAb titers levels at three time-points after vaccination (days 0, 7 and 14) (Table 1), which was higher on day 14, compared to day 7 (Fig. 2a). The percentage of PD-1^+^ICOS^+^ cTfh cells was significantly increased on day 7, compared to that at baseline (*P* < 0.01) (Fig. 2b), and higher than that day on 14. Furthermore, the percentage of CD38^+^CD27^+^CD19^+^ B (plasma) cells was markedly increased on day 7, compared to that at baseline (*P* < 0.05) (Fig. 2c). Expression of miR-92a-1, miR-20a, miR-19a and miR-19b-1 in CD4^+^ T cells was significantly upregulated on day 14, compared to day 7 (*P* < 0.05) (Fig. 2d-g). In addition, both miR-18a and miR-17 levels in CD4^+^ T cells were markedly increased on day 14, compared to day 7 and baseline (*P* < 0.05) (Fig. 2f-h).

**Table 1:** The result of one way repeated measures analysis

	Day 0	Day 7	Day 14	*F*	*P*
HBsAb titers (IU/ml)	57.87 ± 37.93	249.16 ± 125.95	667.39 ± 312.78	48.60	< 0.001
The percentage of PD-1^+^ICOS^+^ cTfh cells	6.17 ± 1.17	7.68 ± 1.62	7.46 ± 2.20	5.62	0.014
The percentage of Plasma cells	20.82 ± 5.03	22.31 ± 6.07	21.66 ± 6.04	2.54	0.114
The expression of miR-92a-1	18.63 ± 0.91	18.35 ± 0.96	19.55 ± 0.76	3.36	0.076
The expression of miR-20a	12.32 ± 0.90	12.01 ± 1.02	13.11 ± 0.68	3.42	0.067
The expression of miR-19a	13.19 ± 1.83	11.11 ± 1.05	12.50 ± 0.75	3.94	0.048
The expression of miR-19b-1	19.18 ± 1.25	18.52 ± 1.06	19.91 ± 0.92	3.37	0.069
The expression of miR-18a	17.84 ± 0.63	17.86 ± 0.96	19.38 ± 0.93	10.37	0.002
The expression of miR-17	10.55 ± 0.70	10.37 ± 0.72	11.44 ± 0.77	5.25	0.023

*F*: The statistics of one way repeated measures analysis

### MiR-18 and miR-17 in CD4^+^ T cells are positively correlated with the PD-1^+^ICOS^+^ cTfh cell population after HB vaccination

**Fig. 3:**
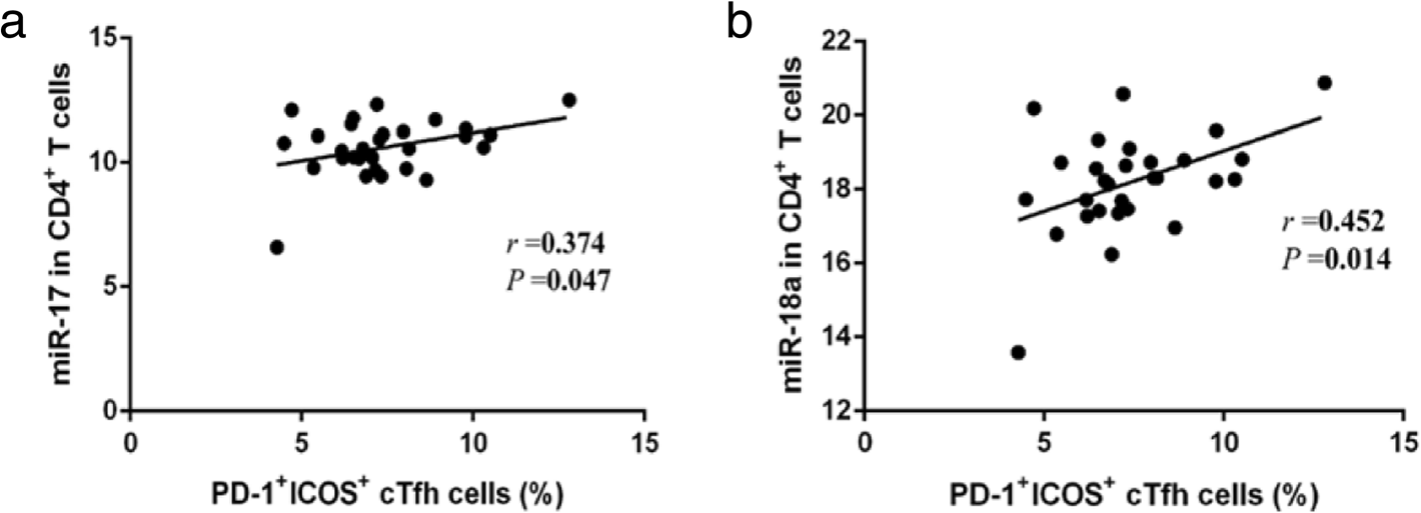
The correlation between PD-1^+^ICOS^+^ cTfh cells and miRNA in CD4^+^ T cells. **a** The correlation of PD-1^+^ICOS^+^ cTfh cells with the level of miR-17 was analyzed. **b** The correlation of PD-1^+^ICOS^+^ cTfh cells with the level of miR-18a was analyzed

Analysis of combined data for all three time-points, revealed that the miR-17 level is correlated with percentage of PD-1^+^ICOS^+^ cTfh cells (*r* = 0.372, *P* = 0.047) (Fig. 3a). Moreover, the miR-18a level in CD4^+^ T cells showed a strong positive correlation with the percentage of PD-1^+^ICOS^+^ cTfh cells (*r* = 0.452, *P* = 0.014) (Fig. 3b). Unexpectedly, the other miRNAs within the cluster failed to show a similar association (*P* > 0.05) (Table 2).

**Table 2:** miR-17~ 92 correlated with the percentage of PD-1^+^ICOS^+^ cTfh cells

	*r*	*P*
PD-1^+^ICOS^+^ cTfh cells and miR-92a-1	0.228	0.254
PD-1^+^ICOS^+^ cTfh cells and miR-20a	0.197	0.306
PD-1^+^ICOS^+^ cTfh cells and miR-19a	−0.022	0.910
PD-1^+^ICOS^+^ cTfh cells and miR-19b-1	0.160	0.408
PD-1^+^ICOS^+^ cTfh cells and miR-18a	0.452	0.014
PD-1^+^ICOS^+^ cTfh cells and miR-17	0.372	0.047

### HBsAb titers are correlated with the PD-1^+^ICOS^+^ cTfh cells population and the miR-17-92 cluster except miR-19a

**Fig. 4:**
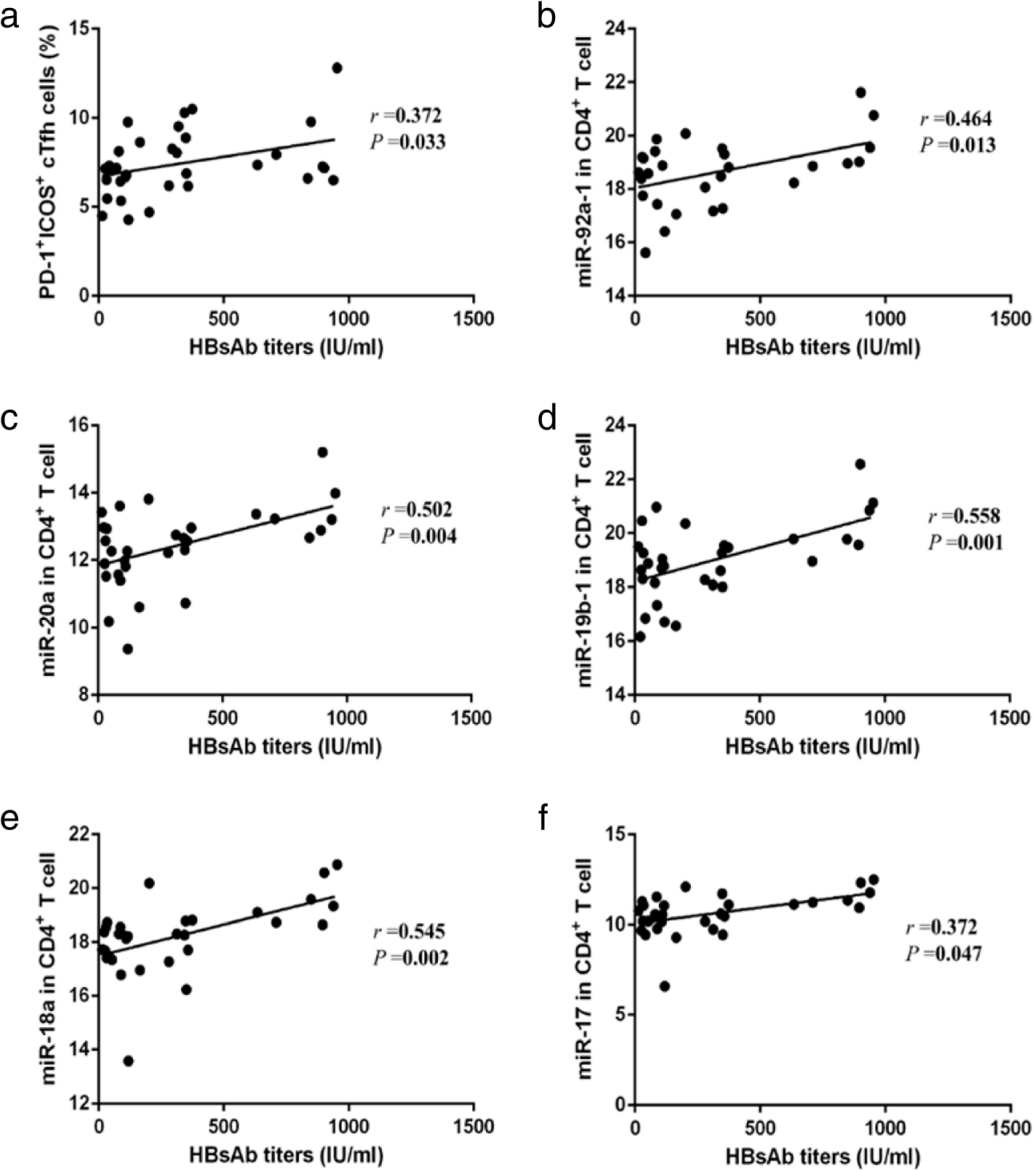
The correlation between the HBsAb titers and PD-1^+^ICOS^+^ cTfh cells, miR17~ 92 in CD4^+^ T cells. **a** The correlation of HBsAb titers with PD-1^+^ICOS^+^ cTfh cells was analyzed. **b** The correlation of HBsAb titers with miR-92a-1was analyzed. **c** The correlation of HBsAb titers with miR-20a was analyzed. **d** The correlation of HBsAb titers with miR-19b-1 was analyzed. **e** The correlation of HBsAb titers with miR-18a was analyzed. **f** The correlation of HBsAb titers with miR-17 was analyzed

Data from the correlation analysis revealed that the HBsAb titer is positively correlated with percentage of PD-1^+^ICOS^+^ cTfh cells (Fig. 4a). Furthermore, increased HBsAb titers are positively correlated with expression of all miRNAs from the miR-17-92 cohort, expect miR-19a (Fig. 4b–f) (Table 3).

**Table 3:** HBsAb titers correlated with cTfh cells and miR-17~ 92

	*r*	*P*
HBsAbtiters and PD-1^+^ICOS^+^cTfh cells	0.372	0.033
HBsAb titers and miR-92a-1	0.464	0.013
HBsAb titers and miR-20a	0.502	0.004
HBsAb titers and miR-19a	0.156	0.401
HBsAb titers and miR-19b-1	0.558	0.001
HBsAb titers and miR-18a	0.545	0.002
HBsAb titers and miR-17	0.492	0.006

## Discussion

Recent studies have reported that blood memory Tfh cells contain three subsets, among which PD-1^+^ICOS^+^ Tfh cells are an activated subset [21, 22]. To our knowledge, this is the first report to explore the potential relationship between the miR-17–92 cohort and PD-1^+^ICOS^+^ cTfh cells after HB vaccination in a Chinese population.

In our experiment, the percentage of PD-1^+^ICOS^+^ cTfh cells was significantly increased on day 7 relative to baseline after HB vaccination (*P* < 0.01). These observations suggest that PD-1^+^ICOS^+^ cTfh cells are involved in the immune response to HB vaccine. Intriguingly, however, emergence of PD-1^+^ICOS^+^ cTfh and plasma cells in blood peaked on day 7 after HB vaccination, suggesting similar kinetics of development for both cell types. Similarly, a recent study showed that emergence of plasmablasts and ICOS^+^CXCR3^+^CXCR5^+^CD4^+^ T cells in blood peaked on day 7 after influenza vaccination [23]. Therefore, induction of these T cells may be an effecting factor for generation of plasma cells and plasmablasts and identification of the pathways or adjuvants that promote their generation is critical.

Another important finding is that the expression of miR-18a and miR-17 in CD4^+^ T cells are positively correlated with the percentage of PD-1^+^ICOS^+^ cTfh cells after HBV vaccination. In combination with previous results, our data suggest that miR-17–92 promotes the differentiation of Tfh cells and maintains the fidelity of cell identity by suppressing non-Tfh cell-related genes both directly and indirectly [15]. The miR-17–92 cluster is reported to positively regulate differentiation of Tfh cells by driving the migration of CD4^+^ T into B cell follicles through modulation of ICOS signaling [24]. MiR-17, as the member of the cluster, it can protected CD4^+^T cells from excessive activation-induced cell death to targeted transforming growth factor-β receptor II(TgfbrII) and cAMP-responsive element-binding protein 1 (Creb1) [25]. Also, the cluster member of miR-18a, it was the most dynamically upregulated microRNA of the miR-17–92 cluster in activated T cells [26]. According, miR-18a and miR-17 may regulate PD-1^+^ICOS^+^ cTfh cell differentiation after HB vaccination.

In addition, we observed a positive correlation between the percentage increase in PD-1^+^ICOS^+^ cTfh cells and HBsAb titers. This finding is consistent with recent data showing that increased generation of ICOS^+^PD-1^+^CXCR3^+^ Tfh cells are positively correlated with induction of protective antibody responses in response to influenza vaccination [11]. Similarly, in a study on seasonal influenza vaccines, the increase in the ICOS+PD-1 + CCR7^lo^ subpopulation of Tfh1 cells were positively correlated with generation of the protective antibody response [27]. These findings clearly suggest that cTfh cells contribute to the generation of antibody responses to HB vaccination.

HBsAb titers were significantly correlated with miR-18a and miR-17 expression in CD4^+^ T cells inour study. Based on the collective results, we propose that miR-18a and miR-17 induce production of HBsAb by regulating cTfh cell differentiation. Furthermore, miR-92a-1, miR-20a and miR-19b-1 were positively correlated with HBsAb titers. In contrast, miR-19a was not associated with the HBsAb titer. Clayton A White et al. have also been shown that miR-19a irrelevant to plasma cell differentiation in vitro [28]. So we suggesting that individual miRNAs interact with each other and exert their functions through regulation of different signaling networks. This finding is consistent with the recent report that overexpression of the miR-17–92 cluster in T cells leads to production of autoantibodies in mice [29]. The combined data support the possibility that miR-17–92 regulates differentiation of cTfh cells and induces the antibody production after HB vaccination.

## Conclusions

Our results provide preliminary evidence that the percentage of PD-1^+^ICOS^+^ cTfh cells are positively correlated with expression of miR-18a and miR-17 in CD4^+^ T cells after HB vaccination, which may aid in the strengthening the rationale for design of improved vaccines. Future studies should focus on establishing the mechanisms by which miR-18a and miR-17 regulate cTfh cell differentiation.

## Methods

### Subjects and samples

The present study was conducted in a Chinese Han population. A cohort of 12 healthy adults was voluntarily recruited from the community health service center of Dalang, Dongguan (Table 4).The HB vaccine (20 μg) was administered via intramuscular injection of the deltoid according to a 0-, 1-, and 6-month standard schedule (recombinant hepatitis B vaccine, Engerix-B, GlaxoSmithKline, Brentford, UK) [30].None of the subjects had a history of infection with HBV, hepatitis C virus or human immunodeficiency virus, and none were immunodeficient. There were no smokers among the study subjects. We administered a booster HB vaccine via intramuscular injection in the upper arm deltoid and collected 25 mL peripheral venous blood at baseline before and after vaccination on days 7 and 14.

**Table 4:** Demographic characteristics of study cohort

*N*	12
Gender (M/F)	6/6
Age (years)	28.83 ± 3.95
BMI	21.81 ± 3.54

### Cell isolation and purification

Peripheral blood mononuclear cells (PBMCs) were isolated by whole blood. Whole blood was diluted with equal volume PRMI Medium 1640 (Life, USA) and then added to a SEPMATE-50 tube containing density gradient (Lymphoprep)medium (StemCell Technologies, Vancouver, BC) and centrifuged at 1200×g for10 min. The top-layer or supernatant is enriched for PBMCs which were collected, washed 2 × with PRMI Medium 1640 [31]. CD4^+^ T cells were isolated from PBMCs using EasySep™ human CD4^+^ T-cell enrichment kit (StemCell Technologies, Vancouver, BC).

### Flow cytometry analysis

All antibodies were purchased from eBioscience (Thermo Fisher Scientific, Massachusetts, USA) and BD Biosciences (New York, USA). PBMCs were incubated with the relevant mouse anti-human antibodies for 30 min, followed by surface staining for the indicated markers. The antibodies used to analyze B cells were PE-labeled mouse anti-human CD19, FITC-labeled mouse anti-human CD27, BV421-labeled mouse anti-human CD38 (BD Biosciences) and those for T cells were PerCP-Cyanine 5.5-labeled anti-human CD4, PE-labeled anti-human CXCR5, eVolve 655-labeled anti-human PD-1 and APC-eFluor 780-labeled anti-human ICOS (eBioscience).

### Antibody assays

Serum was separated for immediate testing of the anti-HBV antibody level using a commercial enzyme-linked immunosorbent assay kit (Da An Gene Co. Ltd., Guangzhou, China).

### RNA isolation and real-time PCR

Total RNA was extracted with TRIzol reagent (Invitrogen, Carlsbad, CA) from CD4^+^ T cells according to the manufacturer’s instructions. Quantitative real-time PCR (qRT-PCR) was applied used to detect expression of mature miR-17–92, and first-strand cDNA generated with the transcriptor first strand cDNA synthesis kit (Roche, Mannheim, Germany) using 2 μg total RNA and miRNA-specific stem-loop reverse transcription primers. The reverse transcription primers for miR-17–92 and U6 small nuclear RNA (snRNAs) are shown in Table 5. QRT-PCR reactions were performed in triplicate in a 96-well plate containing 1 μl of synthesized cDNA, FastStart Universal SYBR Green Master (Roche, Mannheim, Germany) on PikoReal (Thermo Scientific, USA) in a total volume of 10 μL. The reaction procedures were as follows: 95 °C for 10 min, 40 cycles at 95 °C for 15 s and 60 °C for 30 s. The expression levels of miRNAs were normalized to U6 and calculated using the 2^−ΔCt^ method. All of primers were designed and synthesized by Generay Biotechnology (Generay Biotechnology, Shanghai).

**Table 5:** Primers

Gene	Primer sequence (5′ - 3′)
U6 RT	AACGCTTCACGAATTTGCGT
U6 Forward	CTCGCTTCGGCAGCACA
U6 Reverse	AACGCTTCACGAATTTGCGT
miR-17-5p RT	CCTGTTGTCTCCAGCCACAAAAGAGCACAATATT TCAGGAGACAACAGGCTACCTG
miR-17-5p Forward	GCGGCCAAAGTGCTTACAGTG
miR-17-5p Reverse	CAGCCACAAAAGAGCACAAT
miR-18a-5p RT	CCTGTTGTCTCCAGCCACAAAAGAGCACAATATT TCAGGAGACAACAGGCTATCTG
miR-18a-5p Forward	CGGGCTAAGGTGCATCTAGTG
miR-18a-5p Reverse	CAGCCACAAAAAGAGCACAAT
miR-19a-3p RT	CCTGTTGTCTCCAGCCACAAAAGAGCACAATATTT CAGGAGACAACAGGTCTAGTG
miR-19a-3p Forward	CGCCGAGTTTTGCATAGTTG
miR-19a-3p Reverse	CAGCCACAAAAGAGCACAAT
miR-19b-1-5p RT	CCTGTTGTCTCCAGCCACAAAAGAGCACAATATTTC AGGAGACAACAGGGCTGGAT
miR-19b-1-5p Forward	GCGGCAGTTTTGCAGGTTTGC
miR-19b-1-5p Reverse	CAGCCACAAAAGAGCACAAT
miR-20a-5p RT	CCTGTTGTCTCCAGCCACAAAAGAGCACAATATTTCA GGAGACAACAGGCTACCTG
miR-20a-5p Forward	CGGGCTAAAGTGCTTATAGTG
miR-20a-5p Reverse	CAGCCACAAAAGAGCACAAT
miR-92a-1-5p RT	CCTGTTGTCTCCAGCCACAAAAGAGCACAATATTTCAG GAGACAACAGGAGCATTG
miR-92a-1-5p Forward	CGCCGAGGTTGGGATCGGTTG
miR-92a-1-5p Reverse	CAGCCACAAAAGAGCACAAT

### Statistical analysis

Data are presented as means ± SD. One-Way Repeated Measures Analysis of Variance (ANOVA) was applied for comparison of the three groups. For comparison between two populations, paired two-tailed student’s *t* test was performed. Correlations between variables were determined with Pearson’s correlation coefficient. Data were analyzed with SPSS 15.0 (SPSS Inc., Chicago, IL, USA) and GraphPad Prism 5 software (GraphPad Software Inc., La Jolla, CA).The significance level was set at *P* < 0.05 for all statistical analyses.(*, *P* < 0.05; **, *P* < 0.01; ***, *P* < 0.001).

## Abbreviations

(ANOVA): One-way repeated measures analysis of variance
cTfh cell: Circulating Tfh cell
CXCR5: Chemokine receptor 5
GC: Germinal center
HB: Hepatitis B
HBsAb: Hepatitis B surface antibody
HBV: Hepatitis B virus
ICOS: Co-stimulatory molecules
PBMCs: Peripheral blood mononuclear cells
PD-1: Programmed death 1
qRT-PCR: Quantitative real-time PCR
Tfh cell: Follicular helper T cell
Th cell: T helper cell
